# Navigating the landscape of graft ureteral strictures in renal transplant recipients: A single-center case series and literature review

**DOI:** 10.12669/pjms.42.6.15000

**Published:** 2026-06

**Authors:** Asad Bashir, Nasrum Minallah, Fiaz Ahmad Touqeer, Muhammad Mohsin Ayaz, Zia Ul Haq Akram, Ali Asad

**Affiliations:** 1 Asad Bashir, FCPS (Urology) Chairman,Kidney Transplant Surgery, Department of Kidney Transplant Surgery, Pakistan Kidney Liver Institute & Research Center (PKLI&RC), Lahore, Pakistan; 2 Nasrum Minallah, FCPS (Urology) Consultant, Kidney Transplant Surgery, Department of Kidney Transplant Surgery, Pakistan Kidney Liver Institute & Research Center (PKLI&RC), Lahore, Pakistan; 3 Fiaz Ahmad Touqeer, MS (Urology), Consultant, Kidney Transplant Surgery, Department of Kidney Transplant Surgery, Pakistan Kidney Liver Institute & Research Center (PKLI&RC), Lahore, Pakistan; 4 Muhammad Mohsin Ayaz, MBBS, MRCS (UK), FCPS (Urology), CHPE, CRSM, PGD (Sonology), Clinical Fellow, Kidney Transplant Surgery, Department of Kidney Transplant Surgery, Pakistan Kidney Liver Institute & Research Center (PKLI&RC), Lahore, Pakistan; 5 Zia Ul Haq Akram, FCPS (Urology), Department of Kidney Transplant Surgery, Pakistan Kidney Liver Institute & Research Center (PKLI&RC), Lahore, Pakistan; 6 Ali Asad, MBBS, Medical Officer, Department of Kidney Transplant Surgery, Pakistan Kidney Liver Institute & Research Center (PKLI&RC), Lahore, Pakistan

**Keywords:** Balloon dilatation, Renal Transplantation, Surgical revision, Ureteral Stricture

## Abstract

**Background and Objectives:**

Graft ureteral stricture is an uncommon but important complication after renal transplantation that may compromise graft function. Endoscopic balloon dilatation offers a minimally invasive treatment, though regional data are limited. The study aimed to determine the number of endoscopic balloon dilatation procedures required to manage graft ureteral strictures and to evaluate their success rate as a bridge to or alternative to surgical revision.

**Methodology:**

A single-center retrospective case series included 10 recipients treated between May 2018 to August 2025 conducted at the Department of Kidney Transplant Surgery, Pakistan Kidney and Liver Institute & Research Center (PKLI & RC), Lahore, Pakistan, with outcomes assessed based on the number and success of dilatation procedures.

**Results:**

Among 1,070 living-donor renal transplants, graft ureteral strictures occurred in 10 patients, yielding an incidence of 0.93% (95% CI:0.45–1.71%). No ureteral stricture has occurred in our pediatric patients. Recipients were predominantly male (80%) with a median age of 28.50 (IQR:9) years. Most strictures developed within two months post-transplant, presenting as a rise in serum creatinine (median 2.51 (IQR:2.06) mg/dL at diagnosis). The median number of balloon dilatations per patient was 3.50 (IQR: 2). Outcomes included successful resolution in four patients (40%), surgical revision in four patients (40%), and periodic double J stent replacement in two patients (20%). All patients and graft were stable at one year with stable renal function (median serum creatinine 1.60 (IQR:0.44 mg/dL); median eGFR 44.9 (IQR:13.42) mL/min/1.73 m^2^).

**Conclusion:**

Balloon dilatation demonstrated limited long-term success, with only 40% achieving sustained resolution. A majority required surgical revision or ongoing stenting, suggesting its role as a bridging rather than definitive therapy.

## INTRODUCTION

Renal transplantation is the most effective treatment for end-stage renal disease, offering superior survival and quality of life compared to dialysis modalities such as hemodialysis and peritoneal dialysis.^1^,^2^ Urological problems are still a major cause of morbidity after renal transplantation and can jeopardise graft function and patient survival, even with significant advancements in surgical technique and immunosuppressive therapy.^3^ Graft ureteral strictures are one of the more difficult urological complications, with an incidence ranging from 1.4% to 4.7%.^4^

These are frequently ascribed to ischaemic injury from the careful dissection of the ureter blood supply and typically occur at the distal third of the ureter.^5^-^7^ Graft loss, recurrent infections, obstructive nephropathy, and hypertension can all be consequences of an untreated or poorly managed ureteral stricture.^8^ Surgical revision, or ureteroneocystostomy, has historically been the gold standard for definitive management. Although this procedure is effective, it exposes the immunocompromised recipient to the risks of re-operative surgery, including potential damage to the graft vasculature.^9^,^10^ In recent years, balloon dilatation and other minimally invasive endourological techniques have shown promise as a possible first-line substitute. This technique aimed to remove the obstruction without jeopardising the graft and avoiding the risks associated with re-operation.^11^,^12^

Nonetheless, there is still little and conflicting information available about its long-term effectiveness and the best way to use it in a transplant population.^13^ There are still important unanswered questions about the overall effect on graft survival, predictors of success, and the number of dilatation attempts that should be made before stating failure and moving forward with surgery.^14^,^15^ Our single-center experience with graft ureteral strictures in renal transplant recipients is thoroughly examined in the current series. We make an effort to explain the clinical presentation and the outcomes of a structured management approach in which endoscopic balloon dilatation was used as the initial treatment strategy, with repeat dilatations performed in cases of persistent or recurrent obstruction. Surgical revision was considered after failure of multiple dilatation attempts, with an average of approximately three to four procedures required before proceeding to definitive surgical intervention in our cohort.

We hope to offer insightful information that can direct clinical judgement and enhance patient outcomes in the face of this intricate complication by presenting this case series and placing it within an analysis of the body of current literature.

## METHODOLOGY

This was a single-center, retrospective observational case series conducted at the Department of Kidney Transplant Surgery, Pakistan Kidney and Liver Institute & Research Center (PKLI & RC), Lahore, Pakistan.

### Ethical Approval:

The study was initiated after approval from the PKLI Institutional Review Board [PKLI-IRB/AP/00792025; dated: August 15, 2025]. Due to the retrospective nature of the study, the requirement for informed consent was waived.

The study included all patients who underwent living donor renal transplantation at PKLI & RC between 29^th^ May 2018 to 15^th^ August 2025 and who subsequently developed a graft ureteral stricture.

Recipients of living donor kidney transplants performed within PKLI & RC with the diagnosis of a graft ureteral stricture in the post-transplant period were included. None of patients were excluded due to the complete medical record of patients.

A double J stent (size six Fr, length 12 cm) was routinely placed at the time of transplantation and typically removed after approximately three weeks postoperatively. As per institutional protocol, double J stents were maintained for approximately three weeks post-transplant and for six weeks following balloon dilatation. Graft ureteral stricture was defined based on a combination of clinical and radiological criteria. Clinically, patients presented with a rise in serum creatinine from baseline and/or reduced urine output. Radiologically, diagnosis was supported by evidence of hydronephrosis on ultrasonography or confirmation on computed tomography (CT) urography or nephrostogram where indicated. In selected cases, antegrade or retrograde contrast studies were used to confirm the site and severity of obstruction.^16^

Balloon dilatation procedures were performed using either an antegrade approach via percutaneous nephrostomy or a retrograde ureteroscopic approach, depending on anatomical feasibility and operator preference. Procedures were carried out in collaboration between urology and interventional radiology teams. Balloon size length 4 cm, diameter 6 mm, and dilatation was performed under fluoroscopic guidance. Procedures were performed under general or regional anesthesia as appropriate. Percutaneous nephrostomy placement was performed in patients with significant obstruction or impaired renal function prior to definitive intervention. Surgical revision was performed using ureteroneocystostomy, most commonly via the Lich–Gregoir technique, using a single-layer extravesical anastomosis with absorbable sutures.

Patients in whom balloon dilatation failed to achieve sustained clinical and radiological improvement, as defined above, were subsequently considered for surgical revision.^16^ Immediate success was defined as improvement in renal function (decline in serum creatinine) along with radiological resolution or reduction of hydronephrosis without need for further intervention. Failure was defined as persistent obstruction requiring repeat dilatation, surgical revision, or long-term stenting.^17^

Data were extracted from electronic medical records and entered into a standardized database. Patient identifiers were anonymized to ensure confidentiality. The primary outcome measure was to determine the number of endoscopic balloon dilatation procedures required to manage ureteric strictures and to analyze their success rate as a bridge to or alternative to surgical revision.

### Data collected included:

- Demographics include recipient and donor age, sex.
- Transplant details include graft side, ischemia times (cold and warm), anastomosis types: arterial and venous; intraoperative details: ureter count, double J stent stent placement.
- ***Post-transplant course:*** Graft function, double J stent stent dwell time, serum creatinine levels.
- ***Stricture management:*** The quantity of graft explorations, surgical revisions, and balloon dilatations and other procedures.
- ***Outcomes:*** Graft survival and function at one year.

### Statistical Analysis:

IBM SPSS Statistics for Windows, Version 27.0, was used for all statistical analyses. Since the sample size was limited to 10, descriptive statistics were mostly employed. The Shapiro-Wilk test was used to check for distribution normality. The data are presented as a median with IQR for non-normally distributed data or as a mean ± SD for normally distributed data. Categorical variables are summarized as frequencies and percentages (n, %).

## RESULTS

**Fig.1:**
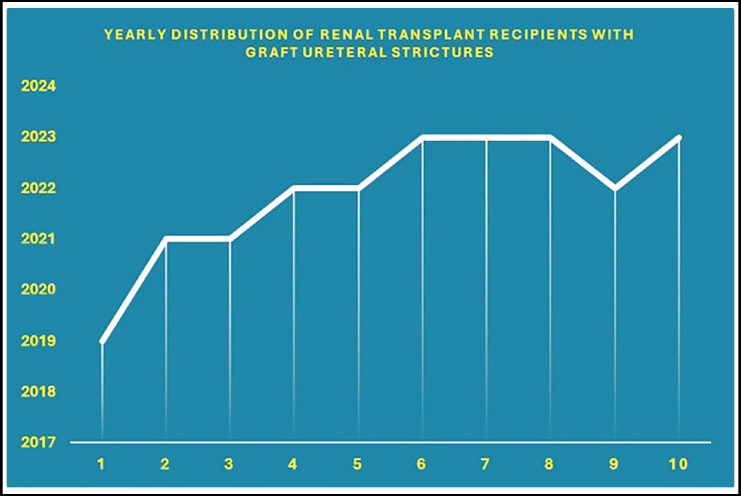
Temporal distribution of renal transplant recipients who developed graft ureteral strictures.

Our center performed 1,070 living-donor renal transplants during the study period, including 44 paediatric recipients (less than 15 years old). Graft ureteral strictures developed in 10 recipients, corresponding to an incidence of 0.93% (95% CI: 0.45–1.71%). There were no cases of graft ureteral strictures in pediatric recipients, and all the observed strictures were in adult recipients. The temporal distribution of these cases across the study period is illustrated in Fig.1.

The baseline characteristics of recipients, donors, and operative parameters are summarized in Table-I. Induction therapy included antithymocyte globulin in 7 (70%), basiliximab in 1 (10%), and no induction in 2 (20%). Immediate graft function was observed in 9 (90%), with 1 (10%) experiencing delayed graft function. No case of urine leakage was reported.

**Table-I:** Recipient, donor, and operative characteristics of patients with graft ureteral strictures (n = 10).

Variable	Categories	n (%) / Median (IQR)
***Recipient Characteristics***		
Age (years)		28.50 (IQR: 9)
Sex	Male	8 (80%)
	Female	2 (20%)
Primary disease	Unknown	3 (30%)
	Antiphospholipid syndrome	2 (20%)
	Nephrolithiasis	1 (10%)
	Primary hyperoxaluria-II	1 (10%)
	IgA nephropathy	1 (10%)
	NSAID-induced nephropathy	1 (10%)
	Posterior urethral valves	1 (10%)
Pre-transplant lower urinary tract reconstruction	Yes	1 (10%)
	No	9 (90%)
***Donor Characteristics***		
Age (years)		35 (IQR: 21)
Sex	Male	8 (80%)
	Female	2 (20%)
***Operative Parameters***		
Side of transplant	Right	8 (80%)
	Left	2 (20%)
Warm ischemia time (seconds)		94 (IQR: 29)
Cold ischemia time (minutes)		55.5 (IQR: 21)
Anastomosis time (minutes)		28 (IQR: 4)
Donor surgery time (minutes)		155 (IQR: 22)
Recipient surgery time (minutes)		255 (IQR: 31)
Graft veins	One	10 (100%)
Graft arteries	One	7 (70%)
	Two	3 (30%)
Graft ureters	One	10 (100%)
Double J stent placement	Yes	10 (100%)

Footnote: Data are presented as mean ± standard deviation (SD) for normally distributed variables, median (interquartile range, IQR) for skewed variables, and frequency (percentage) for categorical variables.

Two patients (20%) required post-transplant exploration for hematoma evacuation. The number of balloon dilatations per patient ranged from one to eight, with a median of 3.50 (IQR: 2). Patients who achieved durable success generally required fewer repeat sessions, whereas those requiring surgical revision underwent a higher number of dilatation attempts, reflecting diminishing returns with repeated endoscopic interventions. The median duration of double J stent placement following transplantation was 21 days (IQR: 1; range: 20–22 days). The duration of double J stent placement prior to balloon dilatation was uniformly 21 days across all patients. Following balloon dilatation, stents were maintained for a period of six weeks. Details of stricture development, diagnostic findings, and management are shown in Table-II.

**Table-II:** Stricture Development, Diagnosis, and Management in Renal Transplant Recipients (n = 10).

Variable	Catagories	n (%),median (IQR)
Stricture Development and Diagnosis		
Dwelling time of double J stent (days)		21 (IQR:1)
Creatinine at double J stent removal (mg/dL)		1.43 (IQR:0.58)
Rise in creatinine after double J stent removal		2.07 (IQR:0.79)
Nadir creatinine (mg/dL)		1.24(IQR: 0.52)
Time to stricture development (days)		58 (IQR:30)
Creatinine at diagnosis of stricture (mg/dL)		2.51(IQR: 2.06)
BK virus positivity at diagnosis	Yes	1 (10%)
	No	9 (90%)
Rejection at diagnosis	Yes	1 (10%)
	No	9 (90%)
Management of Strictures		
Number of balloon dilatations		3.50 (IQR: 2)
Balloon dilatation outcome	Successful	4 (40%)
	Surgical revision	4 (40%)
	Periodic double J stent replacement	2 (20%)

***Footnote:*** Data are presented as mean ± SD for normally distributed variables, median (IQR) for skewed variables, and frequency (%) for categorical variables.

### One-year outcomes:

At one-year, the median serum creatinine was 1.69 (IQR:0.68 mg/dL), with a median eGFR of 44.9 (IQR:13.42) mL/min/1.73 m^2^. While both graft survival (10, 100%) and patient survival (10, 100%) were achieved at one year, one patient who had been planned for surgical revision subsequently developed sepsis, leading to combined graft and patient loss at 29 months post-transplant.

## DISCUSSION

Our series demonstrates that, although uncommon, graft ureteral strictures are a clinically significant complication of living-donor renal transplantation. Indeed, the incidence in our cohort of 0.93% is at the lower end of the range reported in other series, including studies published within the last decade showing incidences that vary from 1.0 to 4.7%.^4^,^18^–^20^ Recipient demographics included a relatively young population with male predominance. Operative parameters were largely within acceptable limits, and only isolated BK virus infections and acute rejection were identified at diagnosis, but strictures still developed, reflecting their multifactorial etiology. No graft ureteral stricture was observed in any of our 44 pediatric transplant recipients, all of whom were under 15 years of age.

Once more, this might indicate a true disparity in risk, or it might just be a result of the fewer paediatric transplants and the shorter follow-up period in general. To ascertain whether these complications actually put paediatric transplant recipients at lower risk, more paediatric transplants and longer follow-up are required. Of the strictures that presented within two months of transplantation, these usually presented as a rise in serum creatinine Balloon dilatation achieved durable success in 4 (40%) patients, while 4 (40%) required surgical revision and 2 (20%) remained dependent on periodic double J stent exchange. These results emphasize the limitations of minimally invasive therapy in refractory cases. Although the periodic exchange of double J stent is not considered standard therapy for graft ureteral stricture, two individuals in our study group opted for this approach. These patients were obese and are currently undergoing attempts at weight loss before undertaking definitive surgical revision. Although periodic exchange of double J stent is suboptimal, it can temporarily serve in highly selected situations in which the risk of surgery is high, or in situations in which the patient desires to delay reconstructive procedures. In patients requiring ongoing stenting, double J stents were maintained for approximately six weeks following balloon dilatation, after which clinical and radiological reassessment was performed. In cases of persistent stricture, repeat balloon dilatation with stent replacement was undertaken. The median requirement of 3–4 balloon dilatation sessions highlights the need for multiple endoscopic interventions before achieving definitive success or proceeding to surgical conversion, reinforcing its role primarily as a bridge rather than a definitive treatment strategy. However, long-term indwelling of stents has risks intrinsic to it, namely infection, encrustation, and lower urinary tract symptoms, once more underlining the fact that there should be a definitive recourse to surgical intervention once the condition of the patient allows. Nonetheless, short-term graft and patient outcomes were superb, with 100% survival and stable renal function at one year, emphasizing the importance of early recognition and tailored intervention.

In our series, similar to previous reports, we found associations with male predominance, multiple renal arteries, delayed graft function, and a previous complex urological history in keeping with risk factors identified by Mahdavi Zafarghandi et al.^18^ and Zhang et al.^19^ However, not all series found strong predictors in both donor and recipient factors, which emphasizes the multifactorial nature of this complication and is comparable to the findings of Minkovich et al.^20^ Three re-examinations were necessary in one of our cases due to postoperative hemorrhage after renal transplantation. It’s possible that numerous surgical operations weakened the ureter’s blood supply, making it more vulnerable to ischemic damage and stricture. Early recurrence of stricture following balloon dilatation may be attributed to underlying ischemia, peri-anastomotic fibrosis, or technical factors during transplantation, which limit the long-term efficacy of endourological interventions. Impaired ureteral blood supply is still a significant risk factor for the development of ischemic stricture, as this case highlights, raising concerns about maintaining periureteral vascularity both during the initial transplant and during subsequent re-explorations.

A minimally invasive approach is reflected in our practice of repeatedly dilatation of the balloon for 40% avoided surgical revision. In contrast to the primarily surgical reconstructions carried out in the Iranian and Chinese series,^18^,^19^ this is in line with the radiological interventions that the Canadian cohort preferred.^20^ The growing arsenal for stricture management is further demonstrated by innovative techniques like magnetic compression anastomosis^19^ and robotic-assisted repairs.^4^

Outcomes across studies remain encouraging, with most series, including ours, reporting preservation of graft function when strictures are recognized and managed promptly. While open repair remains the most durable option for complex strictures,^19^ minimally invasive modalities are increasingly effective, particularly in reducing hospital stay and perioperative morbidity.^4^ Among our patients, four required surgical revision for graft ureteric stricture. Of those, three patients obtained desired outcomes after one revision. However, one patient had a particularly difficult postoperative course. This patient had a history of augmentation ileocystoplasty and bilateral nephroureterectomies, requiring surgical anastomosis of the graft ureter to the augmented segment of the urinary bladder at the time of transplant. In this case, the post-operative course was complicated by recurrent urinary tract infection and later by graft ureteric stricture. This patient required three surgical revisions. The first two surgical revisions were complicated by urinary leakage secondary to inflamed and friable augmented bladder segment. Desired functional outcome was achieved following the third repair.

The present study has a well-characterized cohort with detailed clinical and surgical outcomes, adding strength to the reliability of its findings. However, due to the small sample size and single-center design, it has limited generalizability. Future studies from more centers with larger cohorts and longer follow-up are needed to confirm these observations and better define strategies to optimize the management of post-transplant ureteral stricture.

These findings are put into context by comparing our results with the published literature on graft ureteral strictures (Table-III).

**Table-III:** Comparison of Current Study Findings with Published Literature on Graft Ureteral Strictures in Renal Transplant Recipients.

Study & Setting	Design & Population	Incidence / Prevalence	Reported Risk Factors	Treatment Approaches	Outcomes
Current study (PKLI, 2025)	Case series, n=10 strictures from 1,070 transplants	0.93%	Male predominance (80%), multiple arteries (30%), delayed graft function (10%), prior complex urological history	Balloon dilatation (3.50 (IQR: 2)); 40% success; 40% surgical revision; 20% lifelong stent	No urine leaks, 100% graft and patient survival at 1 year
Siddiqui et al., 2025 (J Endourol) 4	Case series, n=10 robotic repairs	Literature range: 1.4–4.7%	Mostly anastomotic strictures (not detailed)	Robotic repair (ureteroneocystostomy, Boari flap, ureteroureterostomy, ureteropyelostomy)	100% graft function at 3 months; short stay (2 days); 20% complications
Mahdavi Zafarghandi et al., 2013 (Iran) 18	Retrospective, 1,450 RTx; 13 strictures	1.1%	Multiple arteries, cadaveric grafts, prolonged cold ischemia	Open reconstructions (ureteropyelostomy, pyelopyeloplasty)	No recurrence, graft loss, or mortality
Zhang et al., 2023 (China) 19	Case–control, 62 strictures vs 59 controls	Not as %	UTI, DGF, multiple arteries, CMV/BKV infection	Open vs luminal repair vs magnetic compression anastomosis (MCA)	Open = best survival; luminal = highest recurrence; MCA promising (short f/u)
Minkovich et al., 2021 (Canada)^20^	Cohort, 1,742 KTRs	1.2%	No consistent predictors	Radiological (48%) vs surgery (52%)	Strictures ↑ risk of graft failure (HR 3–7) & readmission
Altinel & Acikgoz, 2023 (Turkey) 21	Single-center, 751 live donor KTx	1.0%	Dialysis duration, pre-op urine volume	Not specified (study focused on donor nephrectomy)	All managed successfully; no leaks or graft loss

***Footnotes:*** RTx = Renal transplantation; KTRs = Kidney transplant recipients; UTI = Urinary tract infection; DGF = Delayed graft function; CMV = Cytomegalovirus; BKV = BK virus; MCA = Magnetic compression anastomosis; f/u = follow-up; HR = Hazard ratio.

Incidence values reflect the proportion of stricture cases among total kidney transplantations performed in each study, if available.

Treatment approaches were summarized according to the main management strategies reported in each study.

Outcomes are reported as presented in the original publications and therefore may not be comparable directly, given variation in follow-up duration and definitions.

In agreement with the literature, male preponderance, multiple renal arteries, and delayed graft function were observed in our cohort; however, the small sample size limits definitive inference. Our minimally invasive–first approach is in line with the radiological strategies favored in Canadian series, while surgical reconstructions remain predominant in the studies from Iran and China. More recently, robotic-assisted repairs and magnetic compression anastomosis have widened management options. Despite variation in treatment strategies, most series including ours report favorable graft outcomes when strictures are promptly managed.

### Strengths:

Although the present study has the advantage of case-level characterization, timely intervention, attentive monitoring, and customized management techniques in maximizing long-term outcomes but the single-center design and relatively small sample size limit generalizability.

### Limitations:

Due to the retrospective design, other detailed characterization of strictures (anastomotic vs non-anastomotic, short vs long segment, ischemic vs technical) could not be consistently determined and represents a limitation of this study.

## CONCLUSION

Balloon dilatation shows limited long-term durability in the management of graft ureteral strictures and is often insufficient as a standalone definitive therapy. Most patients ultimately require surgical revision or ongoing stenting, supporting its role primarily as a bridging or temporizing intervention. Early recognition and individualized, stepwise management remain essential to preserve graft function and optimize outcomes.

### Recommendations:

Future studies, preferably multicenter with larger cohorts and longer follow-up periods, are therefore recommended to better delineate risk factors, optimize management pathways, and compare emerging minimally invasive techniques with traditional surgical approaches.

### Author’s Contributions:

**AB:** Conceptualization, supervision, critical revision of the manuscript, and final approval for submission.

**NM:** Data collection, data analysis, and drafting of the manuscript.

**FAT & MMA:** Literature review and interpretation of results.

**ZHA & AA:** Data collection, manuscript editing, and critical revision.

**AB, NM** & **FAT:** Responsible and accountable for the accuracy or integrity of the work.
